# B and T Tumor-Infiltrating Lymphocyte Subtypes According to Subsite: A Colon Cancer Immunophenotyping Map

**DOI:** 10.3390/biomedicines13122856

**Published:** 2025-11-23

**Authors:** Giorgiana Fagarasan, Bogdan Alexandru Gheban, Vlad Fagarasan, Doinita Crisan, Teodora Telecan, Vasile Virgil Bintintan, Radu Ioan Seicean, Alexandra Caziuc, George Calin Dindelegan

**Affiliations:** 1 Department of Anatomy and Embriology, University of Medicine and Pharmacy Iuliu Hatieganu, 400006 Cluj Napoca, Romania; giorgianaamarinei@yahoo.com (G.F.); t.telecan@gmail.com (T.T.); 2 Department of Pathology, County Emergency Clinical Hospital, 400006 Cluj Napoca, Romania; doinitacrisan@gmail.com; 3 Department of Fundamental Sciences, Faculty of Medical Assistance and Health Sciences, University of Medicine and Pharmacy Iuliu Hatieganu, 400006 Cluj Napoca, Romania; 4 Department of Surgery, University First Surgical Clinic, County Emergency Clinical Hospital, University of Medicine and Pharmacy Iuliu Hatieganu, 400006 Cluj Napoca, Romania; vlad.fagarasan@yahoo.com (V.F.); vbintintan@gmail.com (V.V.B.); rseicean@yahoo.com (R.I.S.); alex.8610@gmail.com (A.C.); george.dindelegan@gmail.com (G.C.D.); 5 Discipline of Pathology, University of Medicine and Pharmacy Iuliu Hatieganu, 400006 Cluj Napoca, Romania

**Keywords:** tumor-infiltrating B-lymphocytes, tumor-infiltrating T-lymphocytes, colon cancer, location

## Abstract

**Background:** Accumulating evidence regarding the association between tumor-infiltrating lymphocyte (TIL) subtypes and prognosis in colorectal cancer has emerged recently in the literature. Whether the prognostic impact of TIL subsets is different according to tumor location remains unknown, despite genetic, epigenetic and molecular differences between the proximal and distal colon. Our study aimed to investigate the value of CD3^+^ lymphocytes, reflecting overall T-cell infiltration, CD8^+^ cells identifying cytotoxic effector T-cells and CD73^+^ cells acting as a modulator of immunosuppression, stratified by primary tumor location. **Methods:** The density of CD73^+^, CD3^+^ and CD8^+^ tumor-infiltrating B- and T-cells was determined in colon cancer patients using whole-section tissue sampling, heat-induced epitope retrieval, primary antibodies and DAB visualization. QuPath Cell counter function quantified nucleated cells and immune-positive percentages; ImageJ assessed staining intensity via color deconvolution and optical density. An Immunoreactive Score combined intensity and positivity for immune profiling. The Receiver Operating Characteristic (ROC) curve analysis was used to determine the optimal cut-off values for CD3^+^, CD8^+^ and CD73^+^ lymphocytes. Statistical analysis was performed in order to identify potential associations between TILs expression and pathological characteristics, according to the location of the primary tumor. Survival analysis was carried out using the Kaplan–Meier method. **Results:** A total of 100 patients were included in the study. CD3^+^ T-cells were the most abundantly expressed and were more predominantly encountered in the right colon. Total CD3^+^ numbers were correlated with T stage and the presence of perineural invasion in left-sided tumors, as well as with tumor grading in the right colon. Correlation analysis based on CD3^+^ threshold values according to tumor location demonstrated a statistically significant association between a higher N stage and low CD3^+^ cell values (*p* value = 0.0306), and higher perineural invasion and low CD3^+^ TILs values in the left colon (*p* value = 0.0123). In addition, low CD8^+^ values were associated with a higher T stage in the left colon (*p* value = 0.0382). Survival analysis did not demonstrate statistically significant differences between the investigated groups. **Conclusions:** TIL subtypes in colon cancer patients demonstrate significant variability according to the location of the primary tumor and are associated with different clinical and pathological characteristics. This exploratory study requires larger validation before TIL densities can guide therapy.

## 1. Introduction

Colorectal cancer is the third most common malignant tumor globally in both sexes and the second leading cause of cancer-related death worldwide, despite recent advances in the treatment of this disease [1]. There is an urgent need to identify novel and specific prognostic and predictive targeted biomarkers for colon cancer patients in order to improve patient outcomes. Cancer immunity has recently emerged as a hallmark of tumor biology and the immune response associated with the tumor may inhibit or promote specific mechanisms associated with tumorigenesis, angiogenesis, invasion and even metastasis. Acquired and natural immunity, which is mediated through neutrophils, macrophages and natural killer cells, are responsible for the recognition and destruction of altered or abnormal cells. The immune response is regulated by these cells through specific antibodies which are produced as a response towards tumoral antigens; the activation of cytotoxic lymphocytes leads to the apoptosis of tumor cells. Despite the complexity of these mechanisms, a significant number of colon cancers demonstrate limited immune cell infiltration and are thus defined as “cold tumors”. Numerous cancers have the ability to produce inhibitor factors for T- and B-lymphocytes, enabling tumor cells to proliferate and avoid immunosurveillance mechanisms. Due to this fact, checkpoint inhibitors which increase the immune cells response have been approved as immunotherapy in several types of metastatic cancers [2,3]. Previous studies have demonstrated that the presence and density of various tumor-infiltrating lymphocyte (TIL) subtypes have a significant influence on overall survival, disease-free survival and cancer-specific survival, thus demonstrating prognostic and predictive value as biomarkers for several types of cancers, including colorectal cancer [4,5]. Each subgroup of TILs (T- and B-cells) presents a protein structure on the membranous surface, known as a Cluster of Differentiation (CD), which represents the molecular immune footprint of every T- and B-cell. Recently published data has suggested that colorectal cancer patients whose tumors are highly abundant in CD3^+^ TILs, particularly with Immunoscore values exceeding seven, have been associated with improved cancer-specific survival and overall survival compared to patients with low CD3^+^ infiltrate levels. In addition, high CD8^+^ T-cell values have been associated with prolonged overall survival and cancer-specific survival in colon cancer patients [6,7,8]. More recently, particular attention was given to tumor-infiltrating B-cells in various cancer types. Studies show that tumor-infiltrating CD20^+^ and CD138^+^ B-cells have been associated with favorable outcomes in lung cancer and breast cancer, while in metastatic colorectal cancer, the accumulation of CD20^+^ B-cells has been associated with reduced risk for disease recurrence and improved overall prognosis [9,10,11,12]. In addition, a recently published study which investigated a different subtype of B-lymphocytes, suggested that a high infiltration of CD73^+^ B-cells correlates with improved oncological outcomes in colorectal cancer. CD73 plays an essential role in tumor-mediated immune evasion due to the enzymatic conversion of extracellular adenosine monophosphate into adenosine, a metabolite which inhibits T-cell activation, proliferation and cytokine production, therefore blunting antitumor immune responses, while also promoting angiogenesis, enhancing tumor cell adaptation to hypoxia and facilitating resistance to cellular stress. For this reason, this specific immune tumoral marker is nowadays gaining considerable interest as a potential therapeutic target [13]. Despite these recent insights, colorectal cancer is a heterogeneous disease and the precise functioning of the tumor microenvironment is not sufficiently understood. Differences between proximal and distal colon cancers have been observed, manifesting as particular molecular and genetic characteristics. Immuno-oncology is a relatively new and a rapidly emerging field that has elicited promise in cancer therapy, with several recent studies published on the topic of the immune tumor microenvironment demonstrating the importance of tumor location on prognostic outcomes. The aim of our study was to determine the expression of both B-cell (CD73^+^) and T-cell (CD3^+^ and CD8^+^) subtypes in the colon cancer microenvironment, by establishing the density and prognostic value of these specific subtypes of immune cells according to the location of the primary tumor, in the right or left colon, respectively. A deeper understanding of the variable immune system responses according to tumor location may lead to the development of more accurate targeted immune-modulating therapies [14].

## 2. Materials and Methods

We conducted a longitudinal observational study on a series of consecutive patients diagnosed and treated for colon cancer at First Surgical Department Cluj-Napoca, County Emergency Clinical Hospital, between January 2022 and December 2024. Inclusion criteria for our study were patients diagnosed with stage I to stage III colon adenocarcinoma, who underwent curative surgery in the specified time period. We excluded patients with metastatic colon cancer, non-metastatic rectal cancer, synchronous colorectal cancer or synchronous cancers with other locations, as well as patients who underwent neoadjuvant chemotherapy, or patients with obstructive or perforated colorectal tumors who underwent emergency surgery. We also excluded patients who presented with malignant appendiceal tumors.

Pretreatment staging methods consisted of a colonoscopy with biopsy for tumor histology confirmation and computed tomography for the evaluation of the extent of the disease. The resected specimens were pathologically classified according to the eighth edition of the TNM system. All patients were postoperatively followed up with physical and blood examinations, consisting of periodic measurement of tumor markers levels (CA19-9 and CEA), as well as periodic mandatory postoperative screening using computed tomography and colonoscopy in accordance with ESMO guidelines. We also recorded the location of the primary tumor, age, sex, ASA score, pTNM stage, the number of harvested lymph nodes, the number of positive lymph nodes, lymphatic, venous and perineural invasion, tumor budding score, the presence of mucinous cell histology and tumor grading. All treatment and clinical data were obtained from the medical charts of the patients. The histopathological characteristics of the excised tumors were obtained from the pathological reports. pTNM staging was performed according to the American Joint Committee on Cancer. We defined the right colon as appendix, caecum, ascending and the proximal two thirds of transverse colon, whereas the left colon was defined as the distal third of the transverse colon, left colic flexure, descending and sigmoid colon, according to the hindgut and midgut embryologic origin. Information on vital status and time of death was obtained from the Romanian Death Registry in May 2025.

This research conformed to the provisions of the Helsinki Declaration on ethical principles for Medical Research involving human subjects. All regulations and requirements for handling human samples have been fully complied with during the conduct of our research. Ethical permission for the present study was obtained from the Ethics Committee of University of Medicine and Pharmacy Iuliu Hatieganu, Cluj Napoca (DEP 187/9 June 2023).

Written informed consent has been obtained from each subject upon inclusion in the study.

### 2.1. Tissue Processing and Staining

The paraffin-embedded colon tumor tissue blocks were retrieved from the pathology archives. Sections of 4 μm thickness were cut from each block using a microtome and mounted onto glass slides for Hematoxylin and Eosin (H&E) staining or charged slides for immunohistochemistry (IHC) to ensure tissue adhesion during subsequent processing.

### 2.2. Hematoxylin and Eosin (HE) Staining

For morphological assessment, sections designated for H&E staining were processed using standard histological techniques. The slides were first deparaffinized by immersion in xylene (three changes, 5 min each) to remove the paraffin wax. Sections were then rehydrated through a descending series of graded alcohols (100%, 95%, 70%; 2 min each) and rinsed in distilled water. Nuclear staining was achieved by immersing the sections in hematoxylin solution for 5–10 min, followed by a rinse in running tap water to remove excess stain. Differentiation was performed using 1% acid alcohol (1% HCl in 70% ethanol) for 5–10 s to sharpen nuclear detail, followed by another rinse in tap water. The sections were then blued in a weak alkaline solution for 1–2 min to convert the hematoxylin to a blue hue. Cytoplasmic staining was accomplished by immersing the sections in eosin Y (1% aqueous solution) for 1–2 min. After staining, the sections were dehydrated through an ascending series of alcohols (70%, 95%, 100%; 2 min each), cleared in xylene (two changes, 5 min each), and mounted with a permanent mounting medium under a coverslip.

### 2.3. Immunohistochemical (IHC) Staining

Sections were stained to detect the immune markers CD3, CD8 and CD73. The slides were deparaffinized in xylene (three changes, 5 min each) and rehydrated through graded alcohols (100%, 95%, 70%; 2 min each) to distilled water. Antigen retrieval was performed to unmask epitopes using heat-induced epitope retrieval (HIER). Sections were placed in a citrate buffer (pH 6.0) and heated in a steamer at 95–100 °C for 20 min, followed by a 20 min cooling period at room temperature. Endogenous peroxidase activity was quenched by incubating the sections in 3% hydrogen peroxide for 10 min at room temperature, followed by a rinse in phosphate-buffered saline (PBS). To block non-specific binding, sections were incubated with 10% normal goat serum in PBS for 30 min at room temperature. The sections were then incubated overnight at 4 °C with the following primary antibodies diluted in PBS with 1% bovine serum albumin (BSA):

- Anti-CD3 (rabbit monoclonal, clone SP7, Dako, Glostrup, Denmark) at a 1:100 dilution, to detect T-lymphocytes,
- Anti-CD8 (mouse monoclonal, clone C8/144B, Dako) at a 1:50 dilution, to identify cytotoxic T-cells,
- Anti-CD73 (rabbit polyclonal, Abcam, Cambridge, UK) at a 1:200 dilution, to assess ectonucleotidase expression related to immune regulation.

After incubating with the primary antibody, the sections were washed in PBS three times for 5 min each and incubated with a secondary antibody using the EnVision+ system (Dako, Glostrup, Denmark), which employs a horseradish peroxidase (HRP)-conjugated polymer for signal amplification, for 30 min at room temperature. The immunoreaction was visualized by applying 3,3′-diaminobenzidine (DAB) chromogen for 5 min, producing a brown precipitate at sites of antigen expression. Sections were then counterstained with hematoxylin for 1–2 min, rinsed in tap water, dehydrated through graded alcohols (70%, 95%, 100%; 2 min each), cleared in xylene (two changes, 5 min each), and mounted with a permanent mounting medium.

### 2.4. Slide Scanning and Image Acquisition

All H&E and IHC-stained slides were digitized using a 3D HISTECH Pannoramic scanner (Budapest, Hungary) at 40× magnification to achieve maximum detail suitable for morphometric analysis. This high-resolution scanning produced whole-slide images (WSIs) capturing the entire tissue section. High-definition Tiff images were exported from whole-slide images (WSIs) for each case to enable detailed quantitative analysis, capturing an overall view of the tumor and its adjacent tissues, intra-tumoral regions within the tumor mass, peritumoral regions defined as the area within 500 μm of the tumor edge and stromal areas distant to the tumor beyond the peritumoral region. These specific regions were selected to assess the distribution and proportion of immune-positive cells across the tumor and its microenvironment, facilitating a comprehensive evaluation of immune marker expression in the tissue samples.

### 2.5. Morphometric Analysis

Quantitative analysis of the IHC-stained slides was conducted using QuPath software (version 0.5.1), an open-source platform for digital pathology image analysis, to calculate the percentage of immune-positive cells relative to all nucleated cells in the tumoral and tumor microenvironment regions. To quantify the expression of immune markers CD3, CD8 and CD73 in colon tumor tissue, a systematic procedure was implemented using QuPath software for automated morphometric analysis. Initially, regions of interest (ROIs) encompassing intra-tumoral, peritumoral and distant stromal areas were manually delineated on digital slides by a pathologist to ensure the analysis targeted biologically relevant tissue compartments. Individual cells within these ROIs were identified using QuPath’s ‘Cell detection’ command, which segments cells based on hematoxylin nuclear staining, with detection parameters optimized for colon tumor tissue (e.g., nuclear size: 5–50 μm^2^, background radius: 8 μm, sigma: 1.5 μm) to accurately detect all nucleated cells, including tumor, immune and stromal cells. Cells were then classified as immune-positive or -negative based on DAB staining intensity: for CD3 and CD8, membrane markers, cells were considered positive if the mean DAB optical density in the cell membrane exceeded a predefined threshold, while for CD73, a cytoplasmic marker, positivity was determined by the mean DAB optical density in the cytoplasm surpassing a specific threshold, with thresholds established by analyzing a subset of slides against negative controls and applied uniformly across all samples for consistency.

Finally, the percentage of immune-positive cells was calculated for each ROI using the formula: (number of positive cells/total number of detected cells) × 100, providing a quantitative measure of marker expression across the tumor and its microenvironment. This metric measured the proportion of cells expressing each marker among all nucleated cells in the tumoral and surrounding regions. This automated morphometric analysis using QuPath ensured objective, reproducible quantification of immune cell infiltration CD3, CD8 and CD73 expression, facilitating a comprehensive assessment of the tumor microenvironment in the colon tumor samples. In addition to quantifying the percentage of immune-positive cells, the staining intensity was assessed to provide a more comprehensive evaluation of protein expression. The intensity of DAB (3,3′-diaminobenzidine) staining in immunohistochemistry (IHC) slides was measured using ImageJ (version 1.52a), a free and open-source image analysis software, with color deconvolution in the H-DAB mode. A systematic process was employed using ImageJ software to quantify the staining intensity of DAB in immunohistochemical (IHC) slides. Initially, the “Colour Deconvolution” plugin was utilized to separate the DAB signal, characterized by brown staining, from the hematoxylin counterstain, which appears blue, using predefined H-DAB vectors to isolate the DAB channel corresponding to the target antigen.

The resulting DAB channel image was converted to an 8-bit grayscale format, and the optical density (OD) was calculated using the formula OD = −log10(I/I0), where I represents the pixel intensity and I0 is the background intensity, typically set at 255 for 8-bit images. Mean OD values were then measured within selected regions of interest (ROIs) encompassing cells or tissue areas. Based on these mean OD values, an immune-intensity score ranging from 0 to 5 was assigned, with 0 indicating no staining (OD ≈ 0), 1 representing very weak staining, two denoting weak staining, 3 indicating moderate staining, 4 signifying intense staining, and five corresponding to very strong staining, thereby providing a quantitative assessment of staining intensity.

### 2.6. Immunoscore: Description and Calculation

**Figure 1:**
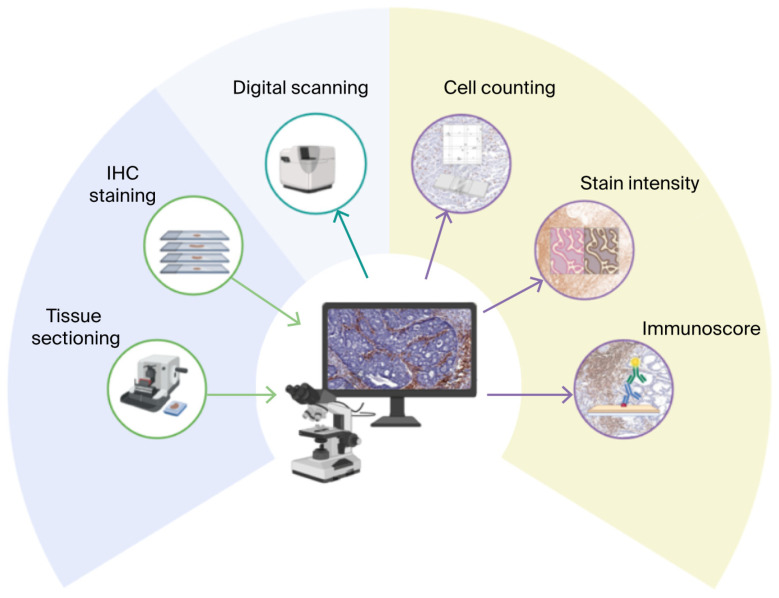
Workflow representation highlighting the tissue sectioning, processing and staining. Microscopical examination of the slides and digital scanning to ensure automated cell counting, stain intensity appreciation and calculation of the Immunoscore.

The Immunoscore concept, originally established and validated by Galon et al., demonstrated that quantifying CD3^+^ and CD8^+^ T-cell densities in the tumor core and invasive margin provides strong prognostic value in colorectal cancer [15]. The Immunoscore is a composite scoring system that integrates staining intensity and the percentage of positive cells to assess immunoreactivity in immunohistochemical (IHC) samples, yielding a score from 0 to 7 that categorizes immunoreactivity as low (1–3), medium (4–5), or high (6–7), with a score of 0 indicating no immunoreactivity. The Intensity Score (I), ranging from 0 to 3, is determined by the staining strength, where 0 represents no staining, 1 indicates weak staining, 2 denotes moderate staining, and 3 signifies intense staining. The Percentage Score (P), ranging from 0 to 4, is based on the proportion of positive cells, with zero assigned to 0% positive cells, 1 to 1–25%, 2 to 26–50%, 3 to 51–75% and 4 to 76–100%. The Immunoscore is calculated by summing the Intensity Score and the Percentage Score, with a maximum possible score of 7 (3 + 4). This standardized approach facilitates consistent evaluation of both the intensity and extent of immune marker expression, enabling reliable comparisons of immune responses across different samples. Since TILs quantification was conducted using digital scanning and automated cell counting, inter-observer variability was not assessed. A schematic representation of the processes involved in establishing the Immunoscore is presented in Figure 1.

### 2.7. Statistical Analysis

Statistical analysis was performed using GraphPad Prism version 9.3.0 for Windows, GraphPad Software, San Diego, CA, USA. Descriptive statistics were calculated as means and percentages for continuous variables. Categorical variables were expressed as contingency tables. Receiver operating characteristic (ROC) curve analysis was used to determine the cut-off value for CD3^+^, CD8^+^ and CD73^+^ T- and B-cells. To ensure an appropriate balance between sensitivity and specificity, we applied the Youden index, defined as Sensitivity + Specificity − 1 in order to maximize overall diagnostic discrimination. The patients were divided into groups according to the specified cut-off values and tumor location. The differences between groups were assessed using Fisher’s exact test and the Mann–Whitney U test for comparisons between continuous and categorical variables. Multivariate analysis of correlation between CD3^+^, CD8^+^ and CD73^+^ TILs and clinicopathological characteristics according to tumor location was performed using the Pearson test. Survival analysis was carried out using the Kaplan–Meier method with log-rank test. Overall Survival was defined as time from initial diagnosis of the tumor until the death of the patient. Disease-free survival (DFS) was defined as time elapsed between surgical treatment and the diagnosis of recurrence on imaging studies. Cox regression proportional hazard models were used to estimate hazard ratios (HR) for death from colon cancer adjusted according to tumor location, using the Immunoscore for CD3^+^, CD8^+^ and CD73^+^ cells as a predictor variable. All statistical tests were two-sided and *p* values < 0.05 were considered statistically significant.

## 3. Results

During the period of time allocated for this study we identified 161 patients with colon cancer. From this cohort, 61 patients were excluded from our study. One hundred patients met the inclusion criteria, had complete data sets and were included for analysis. The demographic data and tumor characteristics of the patients, as evidenced by the histopathological examination, are listed in Table 1.

**Figure 2:**
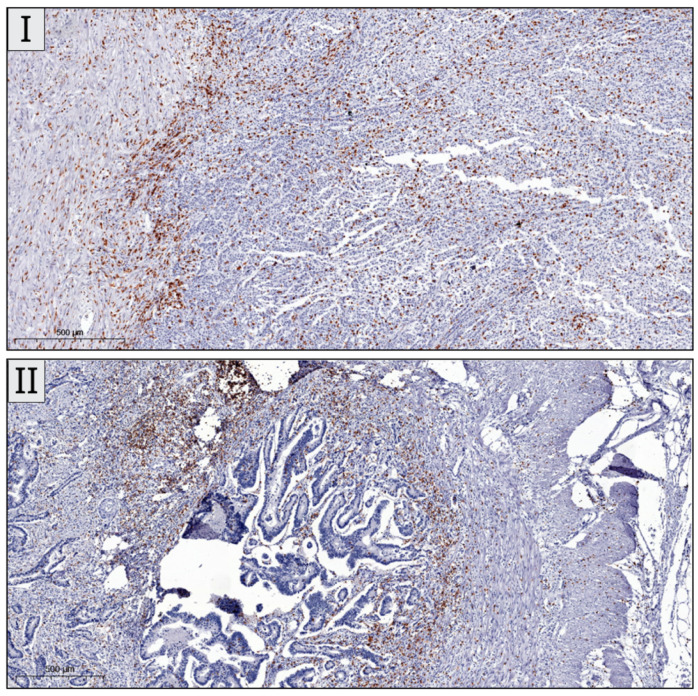

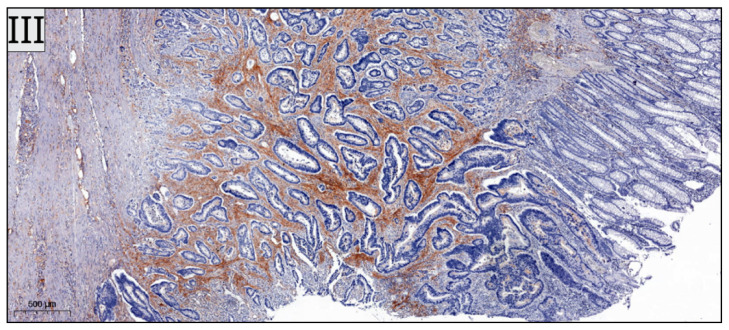
Immunohistochemical images of CD3, CD8 and CD73 staining in colon cancer. (**I**) Presence and spread of CD3 immuno-positive lymphocytes in intra-tumoral and peritumoral areas (4× magnification, CD3 IHC). (**II**) Adenocarcinoma presenting moderate peritumoral CD8 immuno-positive lymphocytes, as well as sparse intra-tumoral lymphocytes (4× magnification, CD8 IHC). (**III**) Immunopositivity of CD73 within the peritumoral lymphocytes surrounding an intestinal type adenocarcinoma (4× magnification, CD73 IHC).

The median age of patients at diagnosis was 69.75 years old, ranging from 42 to 91 years old. In total, 46 patients were female and 54 patients were male. Information on tumor location was available for all the patients, with 49 right-sided colon tumors and 51 left-sided colon tumors. Determination of CD3^+^, CD8^+^ and CD 73^+^ cells was successful in all included cases. Sample IHC images are shown in Figure 2.

CD3^+^ cells were the most abundantly expressed out of the investigated lymphocytes. Overall, TILs were more predominantly encountered in the right colon. The mean number of CD3^+^ T-cells in the right colon was 19.8 (95% CI 13.6–22.2) and in the left colon was 15.66 (95% CI 10–18.1). The mean number of CD8^+^ T-cells in the right colon was 8.855 (95% CI 5.1–10) while the left colon had a mean number of CD8^+^ cells of 7.945 (95% CI 4.1–9.1). For CD73^+^ B-cells the mean number in the right colon was 13,38 (95% CI 9.4–17.1) and in the left colon the mean number was 9.424 (95% CI 5–9.8). Multivariate analysis of correlation between total CD8^+^ and CD73^+^ TILs and pathological characteristics according to tumor location did not demonstrate statistically significant associations. Multivariate analysis of correlation between total CD3^+^ TILs number and pathological characteristics according to tumor location demonstrated significant associations with T stage and perineural invasion in the left colon, as well as between CD3^+^ cells values and tumor grading in the right colon. The results of the analysis are presented in Table 2.

**Figure 3:**
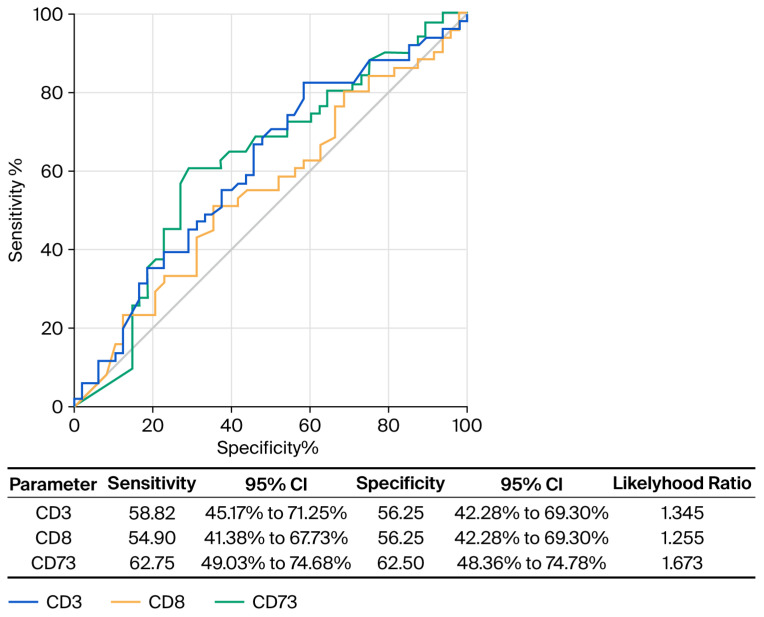
ROC curve analysis of CD3, CD8, CD73.

The optimal threshold values were 15.4 for CD3^+^ (AUC 0.6109, 95% CI 0.4992–0.7226, *p* = 0.0573), 7.05 for CD8^+^ (AUC 0.5502, 95% CI 0.4362–0.6643, *p* = 0.3891) and 9.7 for CD73^+^ (AUC 0.6240, 95% CI 0.5118–0.7362, *p* = 0.0336). The results of the ROC curve analysis are presented in Figure 3.

Based on the established cut-off values, 61.2% of patients with right colon tumors had high CD3^+^ values, while 58.8% of patients with left colon tumors had low CD3^+^ cells values. Statistical analysis demonstrated a significant association between high CD3^+^ TIL values and right-sidedness (OR 0.4345, 95% CI 0.1953–0.9967, *p* = 0.0466). No statistically significant association was observed between CD8^+^ values and tumor sidedness. A total of 60.41% of patients with right-sided colon tumors demonstrated high CD73^+^ values, while 61.53% of patients with left colonic tumors had low CD73 values (OR 0.3685, 95% CI 0.1624–0.8528, *p* = 0.0256).

**Figure 4:**
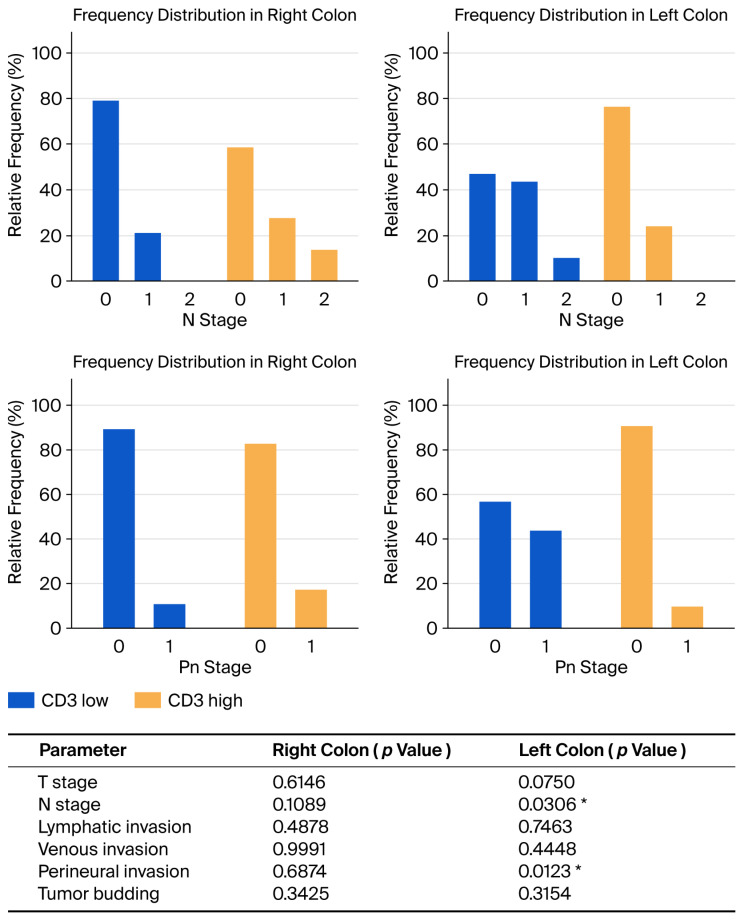
Distribution of cases according to CD3^+^ values and pathological characteristics and results of the statistical analysis according to tumor-sidedness. The top two graphs indicate distribution according to N stage in the right and left colon. The bottom two graphs indicate distribution according to Pn stage in the right and left colon. The * denotes values which are statistically significant (*p* < 0.05).

Correlation analysis between CD3^+^ cells values and pathological characteristics according to tumor location demonstrated a statistically significant association between higher N stage and low CD3^+^ values in the left colon (*p* = 0.0306) and higher perineural invasion and low CD3^+^ values in the left colon (*p* = 0.0123). The results of this analysis are presented in Figure 4.

**Figure 5:**
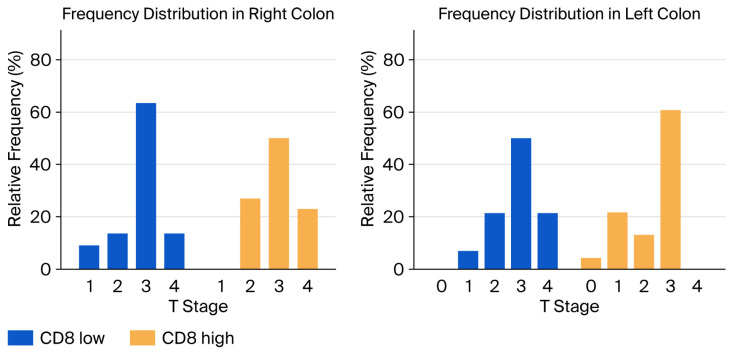
Distribution of cases according to CD8 values and T stage according to tumor-sidedness.

In addition, low CD8 values were associated with a higher T stage in the left colon (*p* = 0.0382). The results of the analysis are presented in Figure 5 and Table 3. No additional correlations were established between CD8 values and tumor location. Lastly, no statistically significant association was established between CD73 values and tumor location.

**Figure 6:**
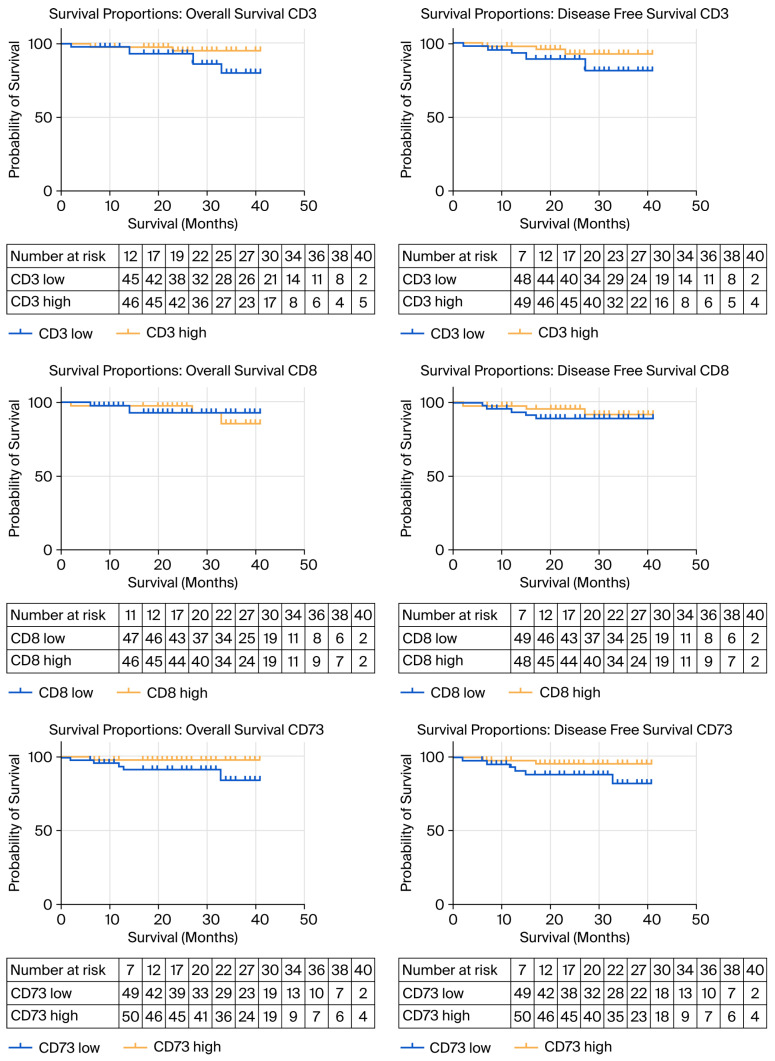
Kaplan–Meier curves for overall survival and disease-free survival according to CD3, CD8 and CD73 cut-off values.

Overall Survival in the entire cohort was 95.94% at one year and 90.69% at three years. Disease-free survival for the entire cohort was 94.81% at one year and 88.53% at three years, respectively. Survival analysis performed according to CD3^+^ cells cut-off values demonstrated increased overall survival (HR 2.754, 95% CI 0.6878–11.02, Chi square 1.708, *p* = 0.1913) and DFS (HR 2.406, 95% CI 0.6964–8.312, Chi square 1.739, *p* = 0.1873) for the CD3^+^ high group, but the differences were not statistically significant. OS and DFS between CD8^+^ high and CD8^+^ low groups was not statistically significant (HR 0.9901, 95% CI 0.1998–4.906, Chi square 0.2632, *p* = 0.6079). Overall Survival and DFS were not statistically significant in the CD73^+^ high group (HR 4.847, 95% CI 0.9781–24.02, Chi square 2.567, *p* = 0.1584, HR 3.047, 95% CI 0.7621–12.19, Chi square 2.080, *p* = 0.1492). Cox proportional hazard regression using the calculated Immunoscore for CD3^+^ as a predictor variable demonstrated a Hazard Ratio of 0,.4498 for OS (95% CI 0.2233–1.119) and 0.647 for DFS (95% CI 0.2008–6.966). When performing Cox regression analysis on cases with tumors located only in the left colon, the HR for DFS was 0.425 (95% CI 0.2117–1.18) for CD3 Immunoscore, 0.5017 (95% CI 0.2456–1.509) for CD8 Immunoscore and 0.642 (95% CI 0.3736–1.217) for CD73 Immunoscore, respectively. The results of the survival analysis are presented in Figure 6.

## 4. Discussion

Several recently published studies have examined the tumor infiltration of T-cells in both rectal and colon cancer patients, attempting to determine the potential prognostic and predictive value of these findings [16,17]. To the best of our knowledge, the present study is the first attempt to investigate the prognostic impact of both T and B immune cell infiltrates in a homogenous group, including only colon cancer patients and emphasizing the anatomical location of the primary tumor. According to the previous multiple proteomic and metabolomic studies, rectal cancer appears to possess particular characteristics which are significantly different from colon cancer, thus necessitating a separate study in order to adequately characterize the tumor microenvironment. The majority of rectal cancer patients also benefit from neoadjuvant chemoradiotherapy, which can influence the density of TILs, thus leading to biased results when analyzing both colon and rectal cancer cases.

A high density of CD73 was not found to be a statistically significant prognostic factor in our study. This finding does not confirm previous observations which indicated that an increased density of the B-cell TILs subtype is associated with a worse prognosis [18]. In light of recent cancer immunology research, we can affirm that even though we did not observe any statistically significant association for CD73^+^ B-cell density, this novel marker should be further investigated in future prospective studies given the fact that CD73 is one of the key enzymes in the signaling pathway of cancers. The expression of CD73 is higher in tumors than in the corresponding healthy tissues and is usually associated with a poorer prognosis. CD73 is a critical factor in the suppression of an adequate anti-tumor immune response, through the regulation of the immune suppressive adenosine, as well as through directly promoting tumorigenesis, metastasis, angiogenesis and cancer cell proliferation. By converting extracellular AMP into adenosine, CD73 generates an immunosuppressive microenvironment that inhibits T-cell proliferation, activation, and cytokine secretion, while promoting regulatory T-cell activity. This immunosuppressive axis not only dampens cytotoxic immune responses but also supports tumor survival, angiogenesis, and metabolic adaptation. Several studies have demonstrated that high CD73 expression correlates with poor prognosis and resistance to immunotherapy in various cancers, including colorectal cancer [19,20]. Therefore, the inhibition of CD73 represents a new and promising approach to increase therapy efficacy [21,22].

The correlation between total CD3^+^ number and pathological characteristics according to tumor location was significantly associated with T stage and perineural invasion in the left colon, as well as with the degree of tumor differentiation in the right colon. These findings are in accordance with previously published research [23]. In addition, our study demonstrated a significant association between CD3^+^ infiltrates and unfavorable tumor characteristics, namely a higher N stage and higher perineural invasion, which were correlated with a reduced CD3^+^ expression in the left colon. Although similar results were observed in a previous study, the total amount of CD3^+^ cells was taken into account and no specific analysis was performed concerning the location of the tumor [24]. Thus, the results presented in our study offer a more accurate characterization of the spatial distribution of lymphocytes compared to previously published research.

The various segments of the colon have a significantly different embryological origin. The proximal part, from the appendix to the first two thirds of the transverse colon, originates from the midgut, whereas the distal part of the transverse colon, the left colic flexure and the remaining colon to the rectum, originates from the hindgut. A relatively large proportion of the studies investigating the differences between right-s and left-sided CRC have used alternative definitions, for example, defining the right colon from the appendix to the hepatic flexure and the left colon from the splenic flexure to the rectum, thus excluding the transverse colon altogether. The definitions present in our study are based on the accepted embryological origin of the respective bowel segments. However, research demonstrates a gradual transition through the multiple anatomic subsites, rather than abrupt changes as in the two-colon model [25]. Nonetheless, as a clinically practicable tool, the two-colon model might still be preferable. The differences extend from an embryological point of view to epidemiological, molecular and genetic characteristics, and thus have the potential to have a different prognosis. Right-sided tumors demonstrate more diverse genetic and molecular characteristics compared to left-sided tumors, and these differences in biological behavior have been suggested to induce variable responses to different types of therapies [26]. The findings from the present study provide further evidence that proximal and distal colon cancer may represent distinct disease entities, wherein the impact of the immune tumor microenvironment on tumor progression, prognosis and prediction can be different. The findings in our study could have a major impact in immunotherapy for patients with loco-regionally advanced colon cancer, and also in developing colon cancer-specific immunotherapy drugs and vaccines according to the specific density of TIL subsets in relation to the site of the cancer (left versus right colon). These novel data challenge the common conception of discrete molecular features of proximal versus distal colon cancers, and have a substantial impact on clinical, translational and epidemiological research, which has typically been performed with the dichotomous, but standard molecular, genetic classification of proximal versus distal tumors.

In our study, a low CD8^+^ T-cell density in the left colon was associated with a higher T stage. This finding is in contrast with a previous study, which demonstrated no difference in CD8^+^ cell infiltration according to tumor subsite [27]. As right-sided colon cancer is demonstrated in some studies to have better survival rates than left sided colon cancer, our findings further emphasize the positive impact of higher densities of CD8^+^ lymphocyte infiltration in right-sided colon cancer [28,29].

The association between low CD3^+^ and CD8^+^ TIL densities and advanced T or N stage is biologically plausible given the central role of effector T-cells in constraining early tumor growth and preventing metastatic spread. Reduced infiltration of total (CD3^+^) and cytotoxic (CD8^+^) T-cells reflects impaired immune surveillance, enabling tumor cells to proliferate and invade with less immunologic pressure. This pattern is particularly relevant in left-sided colon cancers, which are generally characterized by lower baseline immune infiltration and a more immunosuppressive microenvironment. In this context, TIL depletion may further facilitate immune escape by diminishing cytotoxic activity at the invasive margin and weakening antitumor immune control, thereby contributing to more aggressive pathological features. A recently published phase III randomized controlled trial demonstrated that the contribution of TILs infiltration to disease-free survival was substantially greater in right- vs. left-sided tumors (24% vs. 1.5%) [30].

Although our analysis focused on CD3^+^ and CD8^+^ T-cell infiltration, CD4^+^ T-cell involvement represents an additional layer of immune regulation in colorectal cancer. CD4^+^ T-cell subsets can exert both anti-tumor and pro-tumor effects: Th1 cells enhance cytotoxic immunity and are generally associated with favorable outcomes, whereas Th17 cells and regulatory T-cells may promote inflammation-driven tumor progression or suppress effective anti-tumor responses. In contrast, Tfh cells within tertiary lymphoid structures have been linked to improved prognosis [31].

An increased infiltration of lymphocytes has previously been found in MSI-high tumors, with the majority of these being located proximally [32]. This was confirmed in the present study, with the vast majority of right-sided tumors displaying a higher density of CD3+, CD8+ and CD73+ cells. Similar findings have been observed in other studies using the Immunoscore [33]. The correlation between MSI status and TILs is supporting the theory that combined assessment of MSI status and tumor-infiltrating lymphocytes may provide a more accurate prognosis, particularly in patients with right-sided tumors.

The main weakness of the present study is the small number of included patients, which may reduce the overall significance of the statistical results, particularly concerning subgroup analysis. Further studies conducted on larger cohorts of patients are required in order to validate the observed results. In addition, the lack of statistical significance of the survival analysis may be due to the relatively small number of cases included in the study, as well as the relatively short follow-up period. Lastly, the absence of molecular profiling could be considered a limitation of the present study. It should also be pointed out that the prognostic value of B-cells and plasma cells has shown no statistically significant results in our study, so future research should be more focused on the individual effects of B-cell infiltration. Additional future research directions might also be oriented towards the importance of different distribution and density of TILs in specific tumoral subsites in relation with colon cancer vaccines, CAR T-cells therapy and TIL therapy, which are currently being extensively studied [34,35,36,37]. Immunotherapy using different drugs, such as monoclonal antibodies, immune-checkpoint inhibitors, adoptive cell therapy, and also new cancer vaccines, has raised hopes for treating poor-prognosis advanced colorectal cancers that develop resistance to conventional therapies. However, intra-tumor and inter-tumor heterogeneity hinder the success of these treatments. Patients with a similar tumor phenotype may respond differently to the same immunotherapy regimen. Molecular subtyping, mutation-based classifications and not the least, immunoscoring of colon cancer patients, can facilitate the multi-aspect grouping of and improve response to treatment. Personalized immunotherapy using tumor-specific neoantigens offers the opportunity to consider each patient as an individual, deserving of personalized immunotherapy. In the past decade, the development of sequencing and multi-omics techniques has helped us classify patients more precisely, progressing from the quantification of lymphocytes as component biomarkers for the evaluation of sepsis to a more comprehensive characterization of malignant tumors [38]. The expansion of such advanced techniques along with the neoantigen-based immunotherapy could herald a new era in treating heterogeneous tumors such as colon cancer [39]. Quantifying TIL subsets may provide complementary information to existing molecular classifications and Immunoscore-based stratification for immunotherapy. While molecular subtyping identifies intrinsic tumor features, and the Immunoscore captures overall T-cell infiltration, detailed assessment of effector (CD8^+^) and immunosuppressive (CD73^+^) populations can reveal functional imbalances within the tumor microenvironment. Integrating these immunophenotypic data could refine patient selection for checkpoint inhibitors by identifying tumors with high immunosuppressive potential despite moderate overall T-cell density, ultimately improving prediction of therapeutic response and guiding personalized immunotherapy strategies.

## 5. Conclusions

This study provides a novel demonstration of T- and B-cells densities in colon cancer patients according to primary tumor location, demonstrating that TIL subtypes might gradually change along different subsites of the colon. Our findings indicate that tumor location may be an important factor to take into consideration when assessing TILs in colon cancer patients. Different TIL environments may reflect different responses to immunotherapy, highlighting the complexity of the underlying tumor-immune infiltration but also the emerging need for individualized case-based treatment.

## Figures and Tables

**Table 1:** Demographic and tumor specific characteristics of the cohort.

Characteristic	Percentage (%)
Gender	
Male	56
Female	44
Location of primary tumor	
Cecum	19
Ascending colon	24
Transverse colon	13
Descending colon	12
Sigmoid	32
T stage, n	
Tis	1
T1	9
T2	19
T3	56
T4	15
Tumor grade	
G1	30
G2	63
G3	7
Lymphatic invasion	
Negative	79
Positive	21
Venous invasion	
Negative	86
Positive	14
Perineural invasion	
Negative	78
Positive	22
N stage, n	
N0	63
N1a	13
N1b	7
N1c	10
N2a	2
N2b	5
Tumor budding	
Negative	79
Positive	21

**Table 2:** Multivariate analysis of correlation between total CD3^+^ numbers and pathological characteristics according to tumor location.

Parameter	Right Colon			Left Colon		
	R Coefficient	95% CI	*p* Value	R Coefficient	95% CI	*p* Value
T stage	−0.1600	−0.4319 to 0.1386	0.2774	−0.2781	−0.5143 to −0.002735	0.0482 *
N stage	0.07409	−0.2228 to 0.3584	0.6167	−0.2521	−0.4934 to 0.02527	0.0744
Lymphatic invasion	0.1288	−0.1696 to 0.4056	0.3829	0.09699	−0.1835 to 0.3629	0.4984
Vascular invasion	0.07049	−0.2262 to 0.3552	0.6340	−0.08689	−0.3540 to 0.1933	0.5443
Perineural invasion	−0.04900	−0.3362 to 0.2466	0.7408	−0.3522	−0.5723 to −0.08487	0.0113 *
Tumor budding	−0.1290	−0.4057 to 0.1695	0.3823	−0.1118	−0.3758 to 0.1690	0.4347
Degree of differentiation	−0.2980	−0.5428 to −0.006495	0.0397 *	−0.007026	−0.2821 to 0.2691	0.9610

The * denotes values which are statistically significant (*p* < 0.05).

**Table 3:** Correlation analysis between CD8 and CD73 values and pathological characteristics based on tumor location.

TILS	Parameter	Right Colon (*p* Value)	Left Colon (*p* Value)
CD8	T stage	0.7004	0.0382 *
	N stage	0.7919	0.0693
	Lymphatic invasion	0.9777	0.9791
	Venous invasion	0.9999	0.7153
	Perineural invasion	0.8672	0.1253
	Tumor budding	0.4079	0.5095
CD73	T stage	0.5939	0.6444
	N stage	0.3395	0.0603
	Lymphatic invasion	0.7243	0.7261
	Venous invasion	0.6583	0.4502
	Perineural invasion	0.4002	0.8011
	Tumor budding	0.9037	0.7706

The * denotes values which are statistically significant (*p* < 0.05).

## Data Availability

The data presented in this study are available on request from the corresponding author. The data are not publicly available due to privacy reasons.

## Abbreviations

The following abbreviations are used in this manuscript:

TIL	Tumor-infiltrating lymphocyte
CD	Cluster of differentiation
HR	Hazard ratio
IHC	Immunohistochemistry
OS	Overall survival
DFS	Disease-free survival
PBS	Phosphate-buffered saline
HIER	Heat-induced epitope retrieval
